# Genetic divergence analysis of the Common Barn Owl *Tyto alba* (Scopoli, 1769) and the Short-eared Owl *Asio flammeus* (Pontoppidan, 1763) from southern Chile using COI sequence

**DOI:** 10.3897/zookeys.534.5953

**Published:** 2015-11-11

**Authors:** Nelson Colihueque, Alberto Gantz, Jaime Ricardo Rau, Margarita Parraguez

**Affiliations:** 1 Laboratorio de Biología Molecular y Citogenética, Departamento de Ciencias Biológicas y Biodiversidad, Universidad de Los Lagos, Avenida Alcalde Fuchslocher 1305, Casilla 933, Osorno, Chile; 2 Laboratorio de Ecología, Departamento de Ciencias Biológicas y Biodiversidad, Universidad de Los Lagos, Osorno, Chile; 3 Laboratorio de Genética, Acuicultura y Biodiversidad, Universidad de Los Lagos, Osorno, Chile

**Keywords:** Aves, Common Barn Owl, Short-eared Owl, COI, taxonomy

## Abstract

In this paper new mitochondrial COI sequences of Common Barn Owl *Tyto alba* (Scopoli, 1769) and Short-eared Owl *Asio flammeus* (Pontoppidan, 1763) from southern Chile are reported and compared with sequences from other parts of the World. The intraspecific genetic divergence (mean p-distance) was 4.6 to 5.5% for the Common Barn Owl in comparison with specimens from northern Europe and Australasia and 3.1% for the Short-eared Owl with respect to samples from north America, northern Europe and northern Asia. Phylogenetic analyses revealed three distinctive groups for the Common Barn Owl: (*i*) South America (Chile and Argentina) plus Central and North America, (*ii*) northern Europe and (*iii*) Australasia, and two distinctive groups for the Short-eared Owl: (*i*) South America (Chile and Argentina) and (*ii*) north America plus northern Europe and northern Asia. The level of genetic divergence observed in both species exceeds the upper limit of intraspecific comparisons reported previously for Strigiformes. Therefore, this suggests that further research is needed to assess the taxonomic status, particularly for the Chilean populations that, to date, have been identified as belonging to these species through traditional taxonomy.

## Introduction

The Common Barn Owl *Tyto alba* (Scopoli, 1769) (Strigiformes, Tytonidae) and the Short-eared Owl *Asio flammeus* (Pontoppidan, 1763) (Strigiformes, Strigidae) are nocturnal owls usually found in open habitats such as farmland and grassland associated with humanized areas (Weick 2006). The range of both species extends across several continents. In Chile, these owls are sympatric over most of their geographic range, and are considered opportunistic generalists, feeding mainly on small mammals (Rau et al. 1992, Martínez et al. 1998, Figueroa et al. 2009). Although the Common Barn Owl and the Short-eared Owl have been widely studied throughout most of their geographic range in Chile, studies have concentrated mainly on ecological aspects, e.g. diet, conservation and habitat (Raimilla et al. 2012). In contrast, their levels of genetic divergence in comparison with populations in other geographic ranges, have received little attention. This is especially important given that Chilean populations of both species are peripheral populations in the southern hemisphere whose geographic isolation, as a consequence of the Andean mountains that act as a geographic barrier, may have led to evolutionary divergence.

The Common Barn Owl includes several subspecies, some of which may represent distinct and endemic species. To date, up to 25 distinct species have been recognized within the *Tyto* complex but the status of many of these forms remains poorly documented (Weick 2006). Based on morphological data, the Common Barn Owl in Chile has been assigned to the subspecies *Tyto alba tuidara* (J.E. Gray, 1829) (Figueroa et al. 2014). In the Short-eared Owl, nine subspecies have been recognized across its geographic range (Weick 2006), of which the subspecies *Asio flammeus suinda* (Vieillot, 1817) occurs in Chile (Figueroa et al. 2014). Nevertheless, no molecular data supporting the taxonomic identity of the populations of these species in Chile are available yet.

The mitochondrial cytochrome c oxidase subunit I (COI) gene is considered a powerful molecular tool for molecular identification (‘barcoding’) of bird species and may also help to clarify taxonomic relationships (Hebert et al. 2004, Kerr et al. 2007, 2009, Aliabadian et al. 2009). This gene includes a 648-bp region of the mitochondrial genome that provides reasonably good resolution for identifying animal species, as shown by the fact that deep sequence divergences are found between most closely related pairs of animal species (Hebert et al. 2003). Given this property, i.e. its ability to capture species boundaries, this marker has been widely used as a method for identifying species, using a library of sequences linked to taxonomically verified voucher specimens (Ratnasingham and Hebert 2007).

In this study, we sequenced a 648-bp region of the COI gene of the Common Barn Owl and the Short-eared Owl specimens from southern Chile with the aim of comparing these data with the COI sequences published for these species from other geographic areas. Given that no major information on genetic divergence of owls from Chile based on the COI gene is available, this study may contribute to clarifying the taxonomic status and evolutionary divergence at regional and global scales.

## Methods

### Samples collection and DNA extraction

**Figure 1.**
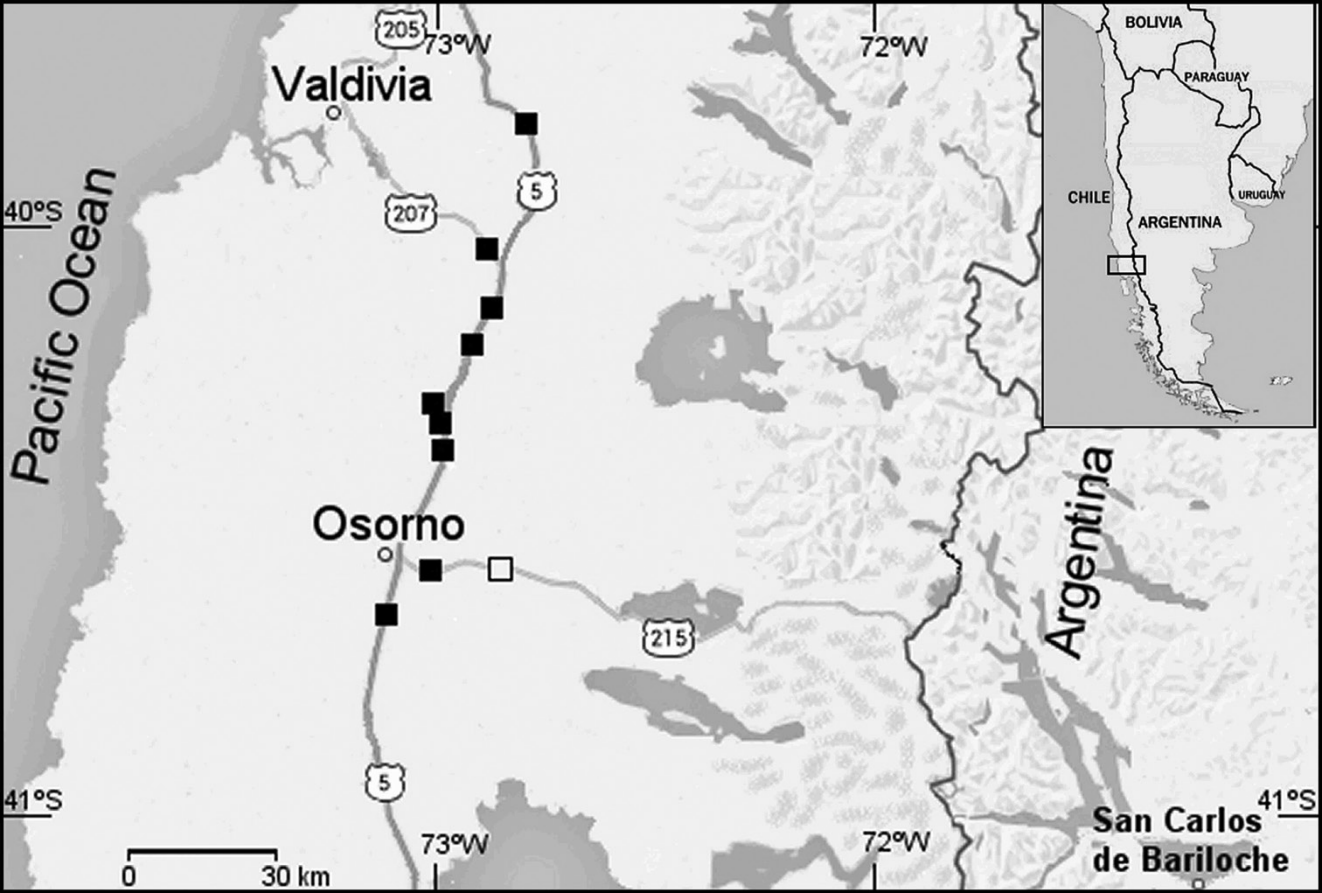
Map of southern Chile, illustrating the collection places of Common Barn Owl and Short-eared Owl specimens. Black squares- Common Barn Owl (n = 9), white square- Short-eared Owl (n = 1).

Owl samples (Common Barn Owl, n = 9; and Short-eared Owl, n = 1) were collected in 2012 and 2013 from dead birds found along the highways (Ruta 5, Ruta 215 and Ruta 207) that connect Osorno with neighboring cities, which run through the provinces of Valdivia, Ranco and Osorno in southern Chile (40°-41° S latitude) (Figure 1). Photographs illustrating the plumage and overall external appearance of Common Barn Owls and Short-eared Owls collected in southern Chile are provided in the Suppl. material 1. DNA was extracted from fixed muscle using the phenol–chloroform method, as described in Taggart et al. (1992). Extracts were standardized at 100 ng/µL using Tris-EDTA buffer pH 8.0.

### PCR and sequencing

COI sequences were amplified using the primer pair of BirdF1 (5’-TTCTCCAACCACAAAGACATT GGCAC-3’) and BirdR1 (5’-ACGTGGGAGATAATTCCA AATCCTG-3’) (Hebert et al. 2004). PCR amplification was carried out in 50 µL using a reaction mix composed of 10 µL Taq polymerase buffer (1 x), 1 µL of dNTPs (0.2 µM), 1.5 µL of MgCl_2_ (1.5 mM), 2 µL of each primer (0.4 µM), 0.5 µL of Taq DNA polymerase (0.05 U/ µL) (Kapa Biosystems), 7.5 µL of template DNA (100 ng/µL), and 25.5 µL of distilled water DNAse, RNAse free (Gibco). The thermal cycling was performed as follows: initial denaturation at 94 °C for 2 min followed by 40 cycles of 94 °C for 45 s, annealing temperature of 58 °C for 45 s, 72 °C for 45 s and a final extension step at 72 °C for 5 min. PCR products were visualized on 2% agarose gels, and prior to sequencing, these were cleaned with QIA quick gel extraction kit (Qiagen). PCR products were bi-directionally sequenced on an Applied Biosystems ABI377 automated sequencer. Sequence records were assembled from forward and reverse reads, and aligned and edited using GENEIOUS 4.0.2 software (Biomatters Ltd.). The sequences are deposited at GenBank (accession numbers KM377628–KM377638).

### Phylogenetic and sequence divergence analyses

Summary statistics, variable and parsimony informative sites, and p-distances were calculated with MEGA 5.05 (Tamura et al. 2011). Relationships among COI haplotypes were reconstructed based on the median joining network implemented in the Network program ver. 4.6.1 (Bandelt et al. 1999). Phylogenetic analyses were carried out using Maximum Likelihood (ML) and Bayesian Inference (BI) approaches. The best-fit nucleotide substitution model was selected using Akaike’s information criterion (AIC). The best model was then used with the ML analyses to construct a ML tree using MEGA 5.05. The consistency of topologies (nodal support) was estimated using a bootstrap approach with 1000 bootstrap replications (Felsenstein 1985). A Bayesian tree was determined using MrBayes v3.1.2 (Huelsenbeck and Ronquist 2001). The analysis was performed using two simultaneous runs of 1,000,000 generations, a random starting tree with four independent Markov chains (MCMC), and tree sampling every 500 generations. The evolutionary model selected for BI analysis was the GTR + Γ + I. The first 25% of the generations were discarded as burn-in, and posterior probabilities were determined by constructing a 50% majority rule consensus for the remaining trees. The trees were visualized using FigTree ver. 1.3.1 (Rambaut 2009).

## Results

### Sequence divergence and phylogenetic relationship of common Barn Owl

**Figure 2.**
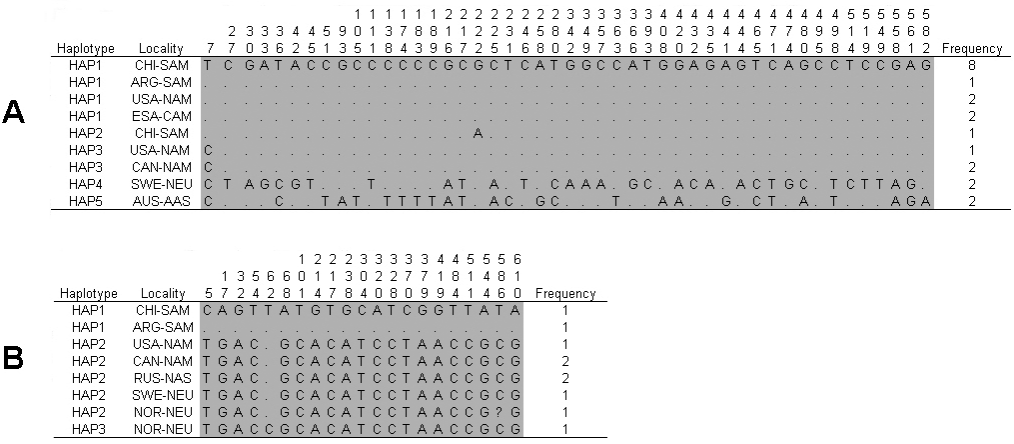
Haplotype designation, variable nucleotide positions of COI gene sequence analysis, with the haplotype frequency observed in each locality. **A** Haplotypes of Common Barn Owls (n = 21) **B** haplotypes of Short-eared Owl (n = 10). Number refers to positions identified in the alignment. For all haplotypes, variable nucleotides are indicated while identity is indicated by slashes.

The sequence alignment of the COI gene obtained had 582 positions in nine specimens of Common Barn Owl from southern Chile, and 12 representative sequences from other regions (one from Argentina, five from North America, two from Central America, two from northern Europe and two from Australasia) obtained from Genbank (http://www.ncbi.nlm.nih.gov/Genbank) (Table 1). For this alignment, five haplotypes, defined by 47 variable positions, were found (Figure 2a). In this alignment, 46 positions were parsimony informative, and 45 out of 47 changes were synonymous. The best fit-model of nucleotide substitution was Tamura-Nei (TN93) with 2165.3 Akaike value and parameter estimates for base frequencies of A = 0.2494, T = 0.2403, C = 0.3292 and G = 0.1810, relative substitution rates of AC = 0.0130, AG = 0.2033, AT = 0.0094, CG = 0.0071 and CT = 0.1846, and proportion of invariables sites of 0.9192.

**Table 1.** List of all owl analysed in this study, with collection localities, coordinates and GenBank accession numbers of COI gene.

Species	Locality (Country/Continent)	Locality abbreviation	Coordinates† (Lat., Long.)	Accession numbers‡
*Tyto alba*	Chile/ South America	CHI-SAM	40.60S, 73.04W	**KM377629**
*Tyto alba*	Chile/ South America	CHI-SAM	40.08S, 72.87W	**KM377630**
*Tyto alba*	Chile/ South America	CHI-SAM	40.29S, 72.97W	**KM377631**
*Tyto alba*	Chile/ South America	CHI-SAM	40.41S, 73.00W	**KM377632**
*Tyto alba*	Chile/ South America	CHI-SAM	40.35S, 72.99W	**KM377633**
*Tyto alba*	Chile/ South America	CHI-SAM	40.29S, 72.94W	**KM377635**
*Tyto alba*	Chile/ South America	CHI-SAM	40.18S, 72.91W	**KM377636**
*Tyto alba*	Chile/ South America	CHI-SAM	39.84S, 72.81W	**KM377637**
*Tyto alba*	Chile/ South America	CHI-SAM	40.69S, 73.13W	**KM377638**
*Tyto alba*	Argentina /South America	ARG-SAM	41.11S, 70.37W	FJ028529
*Tyto alba*	Canada/ North America	CAN-NAM	49.10N, 121.57W	DQ434212
*Tyto alba*	Canada/ North America	CAN-NAM	49.05N, 123.05W	DQ434213
*Tyto alba*	USA/ North America	USA-NAM	25.48N, 80.38W	DQ433249
*Tyto alba*	USA/ North America	USA-NAM	NA	JN850741
*Tyto alba*	USA/ North America	USA-NAM	21.97N, 159.33W	JF498906
*Tyto alba*	El Salvador/ Central America	ESA-CAM	13.44N, 89.05W	KM894402
*Tyto alba*	El Salvador/ Central America	ESA-CAM	13.44N, 89.05W	KM894403
*Tyto alba*	Sweden/ northern Europe	SWE-NEU	55.60N, 13.06E	GU572154
*Tyto alba*	Sweden/ northern Europe	SWE-NEU	55.65N, 13.14E	GU572155
*Tyto alba*	Australia/ Australasia	AUS-AAS	NA	JN801466
*Tyto alba*	Australia/ Australasia	AUS-AAS	NA	JN801467
*Asio flammeus*	Chile/ South America	CHI-SAM	40.60S, 72.88W	**KM377628**
*Asio flammeus*	Argentina/ South America	ARG-SAM	NA	FJ027172
*Asio flammeus*	USA/ North America	USA-NAM	21.31N, 157.95W	JF498831
*Asio flammeus*	Canada/ North America	CAN-NAM	60.43N, 135.04W	DQ433331
*Asio flammeus*	Canada/ North America	CAN-NAM	44.26N, 81.24W	DQ433330
*Asio flammeus*	Sweden/ northern Europe	SWE-NEU	59.20N, 18.12E	GU571744
*Asio flammeus*	Norway/ northern Europe	NOR-NEU	68.49N, 16.67E	GU571269
*Asio flammeus*	Norway/ northern Europe	NOR-NEU	NA	GU571270
*Asio flammeus*	Russia/ northern Asia	RUS-NAS	51.38N,136.55E	GQ481380
*Asio flammeus*	Russia/ northern Asia	RUS-NAS	59.70N, 151.23E	GQ481381

† Coordinates are given in decimal degrees

‡ GenBank Accession numbers in bold were sequenced in this study

**Figure 3.**
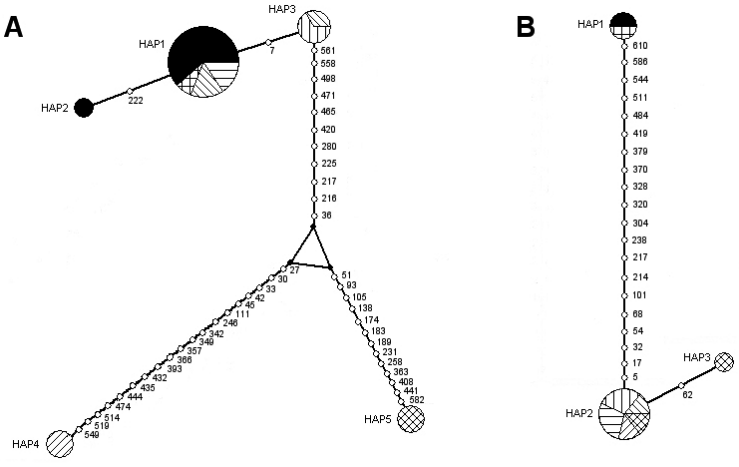
Unrooted haplotype network of COI gene sequence, based on a median-joining network for the Common Barn Owl and the Short-eared Owl. The area of each circle is proportional to the number of individuals containing the haplotype. Open circles indicate mutational events and numbers refer to the variable nucleotide positions. **A** Common Barn Owl; Solid black: Chile/South America; white with cross lines: Argentina/South America; white with forward diagonal lines: USA/ North America; white with horizontal lines: El Salvador/ Central America; white with vertical lines: Canada/ North America; white with backward diagonal lines: Sweden/ northern Europe; white with backward diagonal cross lines: Australia/ Australasia. **B** Short-eared Owl; Solid black: Chile/South America; white with cross lines: Argentina/South America; white with forward diagonal lines: USA/ North America; white with horizontal lines: Canada/ North America; white with backward diagonal lines: Sweden/ northern Europe; white with horizontal lines: Russia/ northern Asia; white with cross diagonal lines: Norway/ northern Europe.

Five haplotypes for the Common Barn Owl specimens were observed, considering all studied localities, with haplotype 1 being exclusive to the New World (including Chile), and representing more than half of the individuals in the data set (61.9%) (Figure 3a). The remaining haplotypes were mostly restricted to a single locality, as were haplotypes 3, 4 and 5, of North America, northern Europe and Australasia, respectively. Of note is that haplotype 2 was exclusive to Chile.

**Figure 4.**
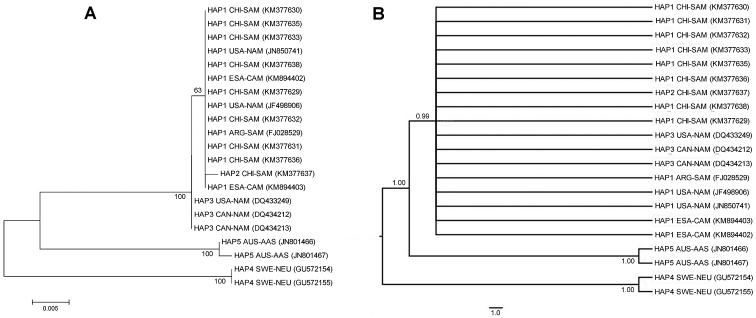
Phylogenetic estimate of relationships among specimens of the Common Barn Owl from different locations based on analysis of 582 bp of the mitochondrial cytochrome c oxidase subunit I gene (COI). The haplotype, locality and Genbank accession number (in parentheses) for each specimen are shown. The branch lengths are drawn proportional to the relative amount of volutionary change. Scale indicates the sequence divergence estimated from the number of nucleotide substitutions per site **A** Maximum likelihood tree with the bootstrap support (%) indicated for each node **B** Bayesian tree with the posterior probability values indicated for each node.

The nine sequences of Common Barn Owl from Chile showed low levels of sequence divergence, ranging from 0 to 0.2% uncorrected p-value. Similarly, genetic divergence between Common Barn Owls from Chile and those from elsewhere in the New World was low, ranging from 0.0 to 0.2%. In contrast, genetic divergence between Common Barn Owls from Chile and those from northern Europe and Australasia was much higher, with p-values ranging from 4.6 to 5.5%. The ML tree showed a cluster of sequences from the New World (including Chile), with a clear separation of sequences from northern Europe and Australasia; this tree had nodes with high (100%) bootstrap values (Figure 4a). The BI tree showed a concordant topology, exhibiting high (0.99–1.00) posterior probability values for each node (Figure 4b).

### Sequence divergence and phylogenetic relationship of Short-eared Owl

The sequence alignment of the COI gene for Short-eared Owl had 611 positions in one specimen from Chile, and nine representative specimens from other regions (one from Argentina, four from North America, two from northern Europe and two from northern Asia) (Table 1). Three haplotypes, defined by 20 variable positions, were identified (Figure 2b). Moreover, in this alignment, 19 positions were parsimony informative, and 17 out of 20 changes were synonymous. The best fit-model of nucleotide substitution was Tamura-Nei (TN93) with 1871.5 Akaike value and parameter estimates for base frequencies of A = 0.2594, T = 0.2507, C = 0.3401 and G = 0.1498, relative substitution rates of AC = 0.0083, AG = 0.2110, AT = 0.0061, CG = 0.0036 and CT = 0.1591, and proportion of invariables sites of 0.9673.

Three haplotypes were found, with haplotype 1 (n = 2 individuals) being exclusive to Chile and Argentina, haplotype 2 (n = 5) exclusive to North America, northern Europe and northern Asia and haplotype 3 (n = 1) exclusive to northern Europe.

COI sequences from Chile and Argentina were identical. However, sequences from Chile/Argentina were highly divergent (3.1%) from those from North America, northern Europe and northern Asia. The ML and BI trees (results not shown) showed one cluster for specimens from Chile and Argentina and another cluster for specimens from North America, northern Europe and northern Asia.

## Discussion

The three major groups of Common Barn Owls inferred here by means of phylogenetic analysis of COI sequences (South America plus North America, Australasia and northern Europe) are similar to those identified in previous phylogenetic studies using other molecular markers (mitochondrial *cyt b* and nuclear *LDHb*) (Wink et al. 2004, Alaie and Aliabadian 2012). In particular, these studies observed a consistent separation between the New World and Old World Common Barn Owls. Moreover, this topology is in accordance with a recent study that used the COI sequence (Nijman and Aliabadian 2013), where strong evidence of a major divergence between New World (*furcata* clade) and Old World (*alba* clade) clades was found. The combined DNA evidence is consistent with the recognition of New and Old World Common Barn Owls as two different species (*Tyto furcata* and *Tyto alba*, respectively) as was originally proposed by König and Weick (2008). However, more extensive studies of morphological, molecular and acoustic divergence among New and Old World populations are highly desirable.

In addition, the genetic distance among the Common Barn Owls from Chile and northern Europe and Australasia, from 4.6 to 5.5%, is not surprising, given that previous studies have also documented high levels of sequence divergence among American and Old World populations of this species, ranging from 5.3 to 7.2% (Alaie and Aliabadian 2012, Nijman and Aliabadian 2013). Therefore, our results support the view that the Common Barn Owl shows strong genetic differentiation across its geographic range, in particular among populations from distant geographic areas.

With regard to the Short-eared Owl, the intraspecific divergence of 3.1% is in accordance with previous studies that also used the COI gene, which reported a maximum divergence of 3.3% for the species (Nijman and Aliabadian 2013) or a mean distance of 3.2% between a single specimen from Argentina and multiple specimens from North America, Europe and northern Asia (Kerr et al. 2009). The divergence between Short-eared Owls from (southern) South America and those sampled elsewhere is interesting and indicates that the taxonomic status of South American populations requires detailed taxonomic study. Again, this should include multiple lines of evidence, including morphology and acoustics, in addition to molecular data.

The levels of sequence variation observed for the Common Barn Owls and the Short-eared Owl from Chile compared to those from other continents was well above the mean intraspecific distance reported for bird species, which ranges from 0.23% to 0.43% (Aliabadian et al. 2009, Hebert et al. 2004, Kerr et al. 2007, 2009). These values are higher than any other owl species analysed so far using COI sequence data, given that in these it has been reported a value of average distance less than 2% (Nijman and Aliabadian 2013).

In previous studies, high levels of sequence divergence have often been interpreted as a possible taxonomic artifact, i.e. as an indication that more taxonomic study is warranted. For instance, Hebert et al. (2004) found four out of 260 species of North American birds with intraspecific divergence ranging from 3.7 to 7.2%, and Kerr et al. (2009), studying the birds of Argentina, observed 13 out of 389 species with sequence divergences exceeding 2.4%. In birds, disjunct ranges, differences in ecology, non-migratory behaviour and rapid speciation may be important factors associated with this phenomenon (Kerr et al. 2009). In the case of the Common Barn Owl, the existence of geographic barriers to gene flow among populations on different continents is to be expected, and this in combination with its non-migratory or short-distance migratory behaviour (Matics 2003), should contribute to promote the genetic divergence. However, all New World Common Barn Owls studied in this work were very similar given that no major genetic divergences were observed. This result agrees with Nijman and Aliabadian (2013), who found no latitudinal clusters for Common Barn Owl sequences from different places of America (North America, the Caribbean, northern South America and southern South America). Although further analysis with other molecular markers is needed to support this result; even so, it is likely that the scarce genetic variation of the Common Barn Owl registered across America may reflect the existence of a particular process of divergence.

In conclusion this study provides further evidence that the Common Barn Owl and the Short-eared Owl show high levels of intraspecific COI sequence divergence across their geographic range. The level of genetic differentiation of both species collected in South America was above the upper limit of species reported for Strigiformes, suggesting that further studies are needed to evaluate the taxonomic position of these populations.

## Supplementary materials

Supplementary material 1 Pictures of external morphology of specimens of *Tyto alba* and *Asio flammeus* collected in southern Chile Data type: Specimens data Explanation note: Pictures of ventral and dorsal views of specimens showing plumage color and overall appearance. Data on body mass of these specimens are also provided. This dataset is made available under the Open Database License (http://opendatacommons.org/licenses/odbl/1.0/). The Open Database License (ODbL) is a license agreement intended to allow users to freely share, modify, and use this Dataset while maintaining this same freedom for others, provided that the original source and author(s) are credited.Nelson Colihueque, Alberto Gantz, Jaime Ricardo Rau, Margarita Parraguez
