# Supplementary material for: Genetic divergence analysis of the Common Barn Owl Tyto alba (Scopoli, 1769) and the Short-eared Owl Asio flammeus (Pontoppidan, 1763) from southern Chile using COI sequence
Source: Zookeys. 2015 Nov 11;(534):135–46. doi: 10.3897/zookeys.534.5953 (PMC4669940; doi:10.3897/zookeys.534.5953)

Supplementary Material I

Pictures of *Tyto alba* and *Asio flammeus* collected in southern Chile.

A-C *Tyto alba*. **A** ventral view, **C** dorsal view. **B-D** *Asio flammeus*. **B** ventral view, **D** dorsal view. Body mass: *Tyto alba* 380 g, *Asio flammeus* 403 g. Scales: **A-D** 10 cm.

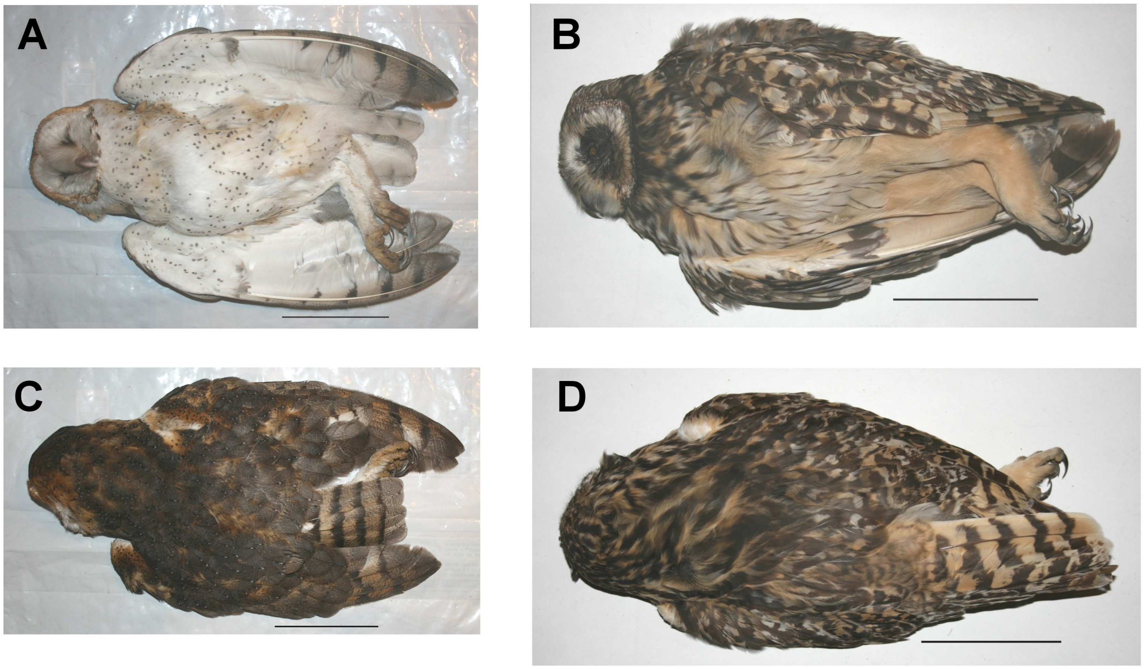

Supplement: Supplementary material 1 — Pictures of external morphology of specimens of Tyto alba and Asio flammeus collected in southern Chile [file zookeys-534-135-s001.pdf]
